# Supplementary material for: Antiretroviral treatment failure and associated factors among people living with HIV on therapy in Homa Bay, Kenya: A retrospective study
Source: PLOS Glob Public Health. 2023 Mar 2;3(3):e0001007. doi: 10.1371/journal.pgph.0001007 (PMC10021395; doi:10.1371/journal.pgph.0001007)
Supplement: Attachment — Submitted filename: Response to Reviewers _9Dec22.docx [file pgph.0001007.s002.docx]

**Response letter to PLOS Global Public Health**

9^th^ December 2022

Dear Editor,

Thank you for reviewing our paper. Please find below a point by point response to the reviewers comments.

Table 1-Journal requirements

|  | Comment | Response |
| --- | --- | --- |
| 1 | Please amend your detailed Financial Disclosure statement. This is published with the article. It must therefore be completed in full sentences and contain the exact wording you wish to be published. | Done |
| 2 | Please clarify all sources of funding (financial or material support) for your study. List the grants (with grant number) or organizations (with url) that supported your study, including funding received from your institution. | Done |
| 3 | State the initials, alongside each funding source, of each author to receive each grant. | Done |
| 4 | State what role the funders took in the study. If the funders had no role in your study, please state: “The funders had no role in study design, data collection and analysis, decision to publish, or preparation of the manuscript.” | Done |
| 5 | If any authors received a salary from any of your funders, please state which authors and which funders. |  |
| 6 | We have noticed that you have uploaded Supporting Information files, but you have not included a list of legends. Please add a full list of legends for your Supporting Information files after the references list. | Done |
| 7 | In the online submission form, you indicated that "The datasets generated and/or analyzed during the current study are presented in the figures and tables in the manuscript. The analytic datasets are available from the corresponding author on request.". All PLOS journals now require all data underlying the findings described in their manuscript to be freely available to other researchers, either 1. In a public repository, 2. Within the manuscript itself, or 3. Uploaded as supplementary information. This policy applies to all data except where public deposition would breach compliance with the protocol approved by your research ethics board. If your data cannot be made publicly available for ethical or legal reasons (e.g., public availability would compromise patient privacy), please explain your reasons by return email and your exemption request will be escalated to the editor for approval. Your exemption request will be handled independently and will not hold up the peer review process, but will need to be resolved should your manuscript be accepted for publication. One of the Editorial team will then be in touch if there are any issues. | Done |

Table2 Editor Comments

| 1 | The analysis does not take into account loss-to-follow-up (i.e. right censoring). Participant data were examined during a 4 month period (Sep 2020 – January 2021). Participants who started ART closer to the end of data collection would, therefore, have lower opportunity for VTF. This could lead to significant bias in classification of suspected TF and, thereby, bias the estimated associations of predictors. Please consider redoing the analysis to account for this bias, such as with time-to-event outcome methods. | We thank the reviewer for this observation. We have redone the analysis using Cox Proportional Hazards modeling to consider right censoring, |
| --- | --- | --- |
| 2 | The definition of VTF is actually those with confirmed elevated VL after EAC. The authors should justify why this outcome is meaningful. It seems that the conservative outcome classification would be to count everyone with suspected TF as VTF unless they have confirmed viral suppression at the post-EAC measure. If the goal is to identify predictors of VTF among those who receive EAC, then the denominator for that analysis should be 280 people who received EAC. | This definition is based on the WHO definition of VTF, and this is the standard used in Kenya. VTF is only confirmed following EAC sessions which addresses any adherence barriers and addressing them and finally repeating a VL. Our goal is to identify factors associated with VTF, this is why our denominator is 160, those who have been diagnosed with VTF |
| 3 | In the Introduction or the Methods, the authors should describe what EAC entails. | We have described this now in the Introduction, line 66-75 |
| 4 | Line 104. Please justify why the study included only those who were on treatment for over 6 months. | We have now provided a justification. This is now line 114-116 |
| 5 | - Line 104. Were all patients who were in the database with treatment over 6 months included in the sample? Please state if other inclusion or exclusion criteria were used to create your sample. | We have included a second inclusion criteria. The clients need to be active on treatment at the time of the study enrolment. This has been included on line 113 |
| 6 | - Line 109. While the timing of the data collection is reported, the range of dates for the retrospective data included in the sample is unclear. Please report the distribution of data with respect to treatment start dates. | This has been addressed on line 121-122. |
| 7 | - Line 124. Please justify why VTF was defined based on VL measurements after 3 months of EAC. | This is based on the WHO/Kenya treatment/EAC guidelines. Clients with VL>=1000copies/ml receive 3 sessions of EAC and a repeat VL is done within 3 months, time within which barriers to adherence should have been addressed .Line 138-139. |
| 8 | - Please report the number of people who had no VL measurements after 3 months of EAC classified. How were these handled in the analysis? | All the 248 participants who received EAC sessions had a repeat VL. Clarification on line 183 |
| 9 | Lines 160-162. I found the % advanced disease reporting confusing. It is reported as 30% in line 160 and then 24.9% in line 163. Is this because the first number included WHO stage III/IV? Or because the second number excluded missing CD4? I suggest deciding on one way to present the % of advanced immune suppression and simply presenting that % only. | Thank you for this comment. Both CD4 and WHO staging was used, where if a patient had a CD4 count that was used, if not the WHO staging was used. The 30% of patients with AHD was based on both CD4 and WHO staging. We have simplified this and reported the % with AHD only based on CD4. Now line 175-176. |
| 10 | - Line 174. The VTF rates are different than Table 3. Please make them consistent. | We have corrected the rates on table 2, line 210 |
| 11 | What was the distribution of time to VTF from EAC? | This has been addressed on line 225-230 |
| 12 | Table 2 contains the same data as Table 3 Column 4, except in column percent vs. row percent. Suggest deleting Table 2. | Table 2 removed |
| 13 | - Line 221. The term “predictor” is used, but some of the variables include those that are likely to be the result of VTF, not antecedent predictors (e.g. CD4 <200 cells/ml). Please justify the inclusion of each variable as “predictor” in the final multivariable model from a conceptual perspective, while avoiding over-adjustment bias. (See Schisterman EF, Cole SR, Platt RW. Overadjustment Bias and Unnecessary Adjustment in Epidemiologic Studies: Epidemiology. 2009 Jul;20(4):488–95.) | Rephrased to: “factors associated with VTF” to clarify that not all are predictors of VTF. Line 232. |
| 14 | Discussion. When providing context with prior studies, please explain how those studies defined VTF and whether they are comparable to the definition used in this manuscript. | Added definitions used of VTF in other studies. Line 256-258. |
| 15 | Line 254. Please explain the usefulness of noting the association between higher VLs and VTF, since VTF is defined by high VLs. | Added this explanation on line 273-275 |
| 16 | Lines 267-268. I don’t believe EAC completion was presented in Results. Please include these data in the Results and use the Discussion to explain the context and implications of those results. | The EAC completion information was only analysed for the 160 participants with VTF, This has now been presented on line 186-187. |
| 17 | Lines 274-275. Please note that low CD4 counts could be the result of the VTF, not the cause of it. Please explain whether the study design allowed for the conclusions that low CD4 may have a causal association (i.e. “predictor”) with VTF. | Agreed that low CD4 counts could be the result of VTF. However, it is no longer a significant factor in the model |

Table 3-Reviewer1 Comments

|  | Comments | Responses |
| --- | --- | --- |
|  | Abstract |  |
| 1 | Line 24: Kindly state which region are referring to. | Region has been added on line 24 |
| 2 | Line 36-37: Be consistent in your reporting. Kindly refer to line 35 (reporting proportion with corresponding 95% CIs). Consider removing these 95% CI relative to proportions given they do not read well. | The 95% CI for the proportions have all been removed. This on line 36-38 |
| 3 | Line 38-40: It is not clear when these CD4 counts were measured. I guess these are CD4 counts at ART-initiation. If so then explicitly state (Patients who initiated ART with CD4 counts < 200 cells/mL were 2.41 times). In addition, add their corresponding 95% CIs as the reader needs to assess the level of significance and the direction of the association. | We have reanalyzed the data using cox-regression and these results are no longer relevant so we have deleted. |
| 4 | Line 44: I fail to understand this part of your conclusion. In fact, HIV program implementers and Health care workers should focus on PLHIV with these characteristics in order to prevent and for early identification of ART treatment failure. | We have revised the conclusion to focus on risk of VTF, on line 43-44 |
| 5 |  |  |
|  | Background |  |
| 6 | Line 48-50: Kindly verify these statistics. These are estimates are for 2020 and the reporting time is mixed up. Updates these statistics with 2021. Kindly see the link below: <https://www.unaids.org/en/resources/fact-sheet> | Thank you. Statistics updated on line 49-52 |
| 7 | Line 52: These figures are obsolete and outdated, kindly update these using the link provided in the comment above. | The figures have been updated on line 53-56 |
| 8 | Line 83-85: While the second part of the study aim is clear, and it has been adequately addressed throughout this paper. However, I have substantial concern regarding the following: “To better describe the HIV treatment cascade”. This was not addressed in this study. | We have removed reference to the treatment cascade, on line 91 |
|  | Methods |  |
| 9 | Line 88-89: Provide further details on the study design, especially on the direction of inquiry. | More details have been provided, line 95-98 |
| 10 | Line 106-107: Is this a desired sample size based on sample size calculation or a sample size chosen arbitrarily (conveniently) based on resource and time. Kindly clarify | We have revised the sample size statement by including the word “convenient”: “We aimed for a convenient sample size of 2,007, which was considered feasible and achievable given the time and resources available.” On line 117 |
| 11 | Line 110-111: It is important to provide further details on how the random systematic sampling was conducted leading to a selection of patients included in the analysis. A single sentence will suffice | We have provided more details: “In each facility, systematic sampling was used to select patient files for abstraction, where every nth record (for example, every 10^th^ file), was selected until the sample size allotted for that facility was obtained. The starting point was obtained by randomly sampling a number between 1 and n, i.e., 1 and 10.”line 123-126 |
| 12 | Line 129-131: Your primary outcome is time-to-event data, reason why Kaplan-Meier method was used to estimate the probabilities of VTF. However, using logistic regression to identify factors associated with VTF is inappropriate for time-to-event data, given logistic regression provides biased estimates when dealing with time-to-event data. To identify factors associated with VTF, Cox-regression methods also known as proportional hazards regression is the suitable/adequate method for the type of data. Kindly re-run these models using Cox-regression methods. | Thank you very much for this comment. We have accepted this suggestion and re-run the analysis using Cox regression. Line 143-146 |
|  | Results |  |
| 13 | Line 147-148: I am not knowledgeable of the of Kenyan context, do you mean individuals aged 0-14 were married?. | Based on the records, these children/adolescents were married/living with partner. In the Kenyan context, these could be children/adolescent who are pregnant/have delivered and living with sexual partners and may report as being married. Line 163 |
| 14 | Line 161: Which method did you use to assess advanced HIV disease ( CD4 count or WHO staging system) ? Looking at table 1, 30% of patients had AHD and this is based on CD4 count. If this is the case, then delete WHO stage III and IV in the sentence | Both CD4 and WHO staging was used, where if a patient had a CD4 count that was used, if not the WHO staging was used. The 30% of patients with AHD was based on both CD4 and WHO staging. we have simplified this and reported AHD based on CD4. Line 175-176 |
| 15 | Line 178: This is not good practice for reporting, DO NOT report mean with 95% confidence intervals, instead report: 1. Mean with standard deviation (SD). 2. Median with IQR (as you have been reporting) On the lighter note, generally, time-to-event data are skewed or asymmetrical (not all the time), kindly check the distribution of your data before reporting the mean time to VTF, otherwise use median time to VTF. | Thank you for this correction. We have modified this to report medians and IQRs. Line 192-194 |
| 16 | Line 220: Refer to my comment in line 129-131. The whole section will change in terms of interpreting findings. I am not expecting substantive changes in the overall results or key findings. There is a sizable number of papers to why Cox proportional hazards models are more suitable instead logistic regression models in when dealing with time-to-event data.  <https://www.nature.com/articles/ejhg200859> | Thank you very much, we have re-analyzed using Cox regression. Line 231 |
|  |  |  |
|  | Discussion |  |
| 17 | Line 237-239: This is critical part of the discussion section as it summarises key findings of the paper. Kindly add VTF was significantly higher among PLHIV who stared ART with CD4 <200 cells/mL. | We have added this statement. There was a difference, but it was not statistically significant. Line 250-253 |
| 18 | Line 247: Change population observed to the "populations studied". Furthermore, the variation observed between your study and Kadima et al. could be partly explained by differences in age cut-off for children. | We have changed to “populations studied". Line 262-264 |
| 19 | Line 258-259: Additional innovative strategies to address sub-optimal adherence to ART among adolescents would be establishment of youth-targeted services, exclusively to youth. <https://onlinelibrary.wiley.com/doi/full/10.1002/jia2.25854> | Thank you! We have added this.  Line 279-281 |
| 20 | Line 269: I suggest adding a more recent evidence from South Africa. <https://onlinelibrary.wiley.com/doi/full/10.1002/jia2.25854> | We have added the more recent evidence. Line 281 |
| 21 | Line 298: What is the possible impact of missing CD4 count data on main findings? Missing data often leads to under or over-estimation (could bias your estimates) of the measures of effects (in the context of your study, this could lead an over or under-estimation of the association between CD4 count and VTF) | We totally agree with your comment, we have added this statement. Line 325-326 |
| 22 | Line 303-304: Consider to add another strength: "Our study was conducted under programmatic conditions (real-world conditions) using routine medical records at public health facilities, demonstrating the burden of VTF in real-world conditions, therefore, maximizing generalizability". | Thank you. We have added this strength. Line 331-334 |

Thank you very much.

Sincerely, Rose Otieno Masaba.
