# Supplementary material for: Antiretroviral treatment failure and associated factors among people living with HIV on therapy in Homa Bay, Kenya: A retrospective study
Source: PLOS Glob Public Health. 2023 Mar 2;3(3):e0001007. doi: 10.1371/journal.pgph.0001007 (PMC10021395; doi:10.1371/journal.pgph.0001007)
Supplement: Attachment — Submitted filename: RESPON~3.DOC [file pgph.0001007.s003.DOC]

**Response letter to PLOS Global Public Health**

23^rd^ January 2023

Dear Editor,

Thank you for reviewing our paper once again. We appreciate the reviewer response and comments raised. Please find below a point by point response to the additional reviewer’s comments.

**Journal Requirements**

| 1 | Please review your reference list to ensure that it is complete and correct. If you have cited papers that have been retracted, please include the rationale for doing so in the manuscript text, or remove these references and replace them with relevant current references. Any changes to the reference list should be mentioned in the rebuttal letter that accompanies your revised manuscript. If you need to cite a retracted article, indicate the article’s retracted status in the References list and also include a citation and full reference for the retraction notice | Thank you for the comment. We have reviewed the reference list. Papers that were not correctly cited have now been corrected. We have not cited papers that have been retracted |
| --- | --- | --- |
| 2 | We have noticed that you have uploaded Supporting Information files, but you have not included a list of legends. Please add a full list of legends for your Supporting Information files after the references list | Thank you for this comment. We have noted that there were legends included for the figures initially but not for the tables listed in the list of legends. We have gone ahead and included the legends for each of the tables in the list of legends and also included the dataset legend |
| Reviewer Comments | | |
| 1 | Line 193-194: Figure 3 compares survival time between patients aged 0-14 and 15+ years and younger patients had shorted time to event. Good.  A Log-Rank test should be used to compare the survival curves, if this was the case, then authors should clearly state in the “statistical analysis section” that “Kaplan-Meier analysis was performed to estimate cumulative incidence of VTF following ART initiation and Log-rank test was used to assess difference in time to VTF between age groups”. Is p=0.0431 resulted from the Log-rank test? | Thank you for this comment. We have addressed this comment and inserted the proposed sentence in the statistical analysis section under line 147-149. We also confirm that the p-value indicated is from the Log-rank test |
| 2 | Line 208-211: there seems to be a confusion in the interpretation of table 2. This table compares demographic and clinical characteristics differences between patients with VL <100 copies vs those with confirmed VL> 1000 copies/ml and chi-square test and corresponding p-values should not be interpreted that way. For instance, for age at ART initiation, the finding means there is a statistically significant difference in age groups between patients with VL <1000 copies vs those with confirmed VL > 1000 copies/ml. Furthermore, table 2 does not seek to assess factors associated with VL suppression and non-suppression, the interpretation thereof is incorrect and misleading. | Thank you for the comment. We have reviewed and confirmed that the goal of Table 2 is not to present the factors associated with VTF. We have updated the table (Table 2) accordingly and updated the text too. The table footers have also been updated |
| 3 | Line 336-337: the conclusion section still needs significant improvement. The authors should expand a bit their concluding statement reflecting the implication of these findings for practice and policy. | We have expanded our conclusion to reflect the implications of these findings for practice and policy. This is in line 342-346 |

Thank you.

Rose Otieno Masaba
